# Our National Almshouses

**Published:** 1899-02-25

**Authors:** 


### The Hospital

A Journal of

The Medical Sciences and Hospital Administration.

Edited by Sir Henry Burdett, K.C.B., and Solomon C. Smith, M.D., M.R.C.P.

Vol. XXV.—No. 648.] [Feb. 25, 1899.

### OUR NATIONAL ALMSHOUSES.

The Central Poor Law Conference, which met last week at the Guildhall, under the presidency of Lord Beauchamp, discussed the question of old age pensions, and in so doing had the undoubted advantage of hearing speakers who took sufficiently opposite views to make the discussion fairly representative. While on the one hand it was maintained that with wages at their present level there were arithmetical limits which made "thrift" an impossible solution of the misery of a poverty-stricken old age, it was on the other hand contended that working-men could afford to provide for their old age, and that the majority of them did so even now. Mr. T. W. Russell, M.P., the Secretary to the Local Government Board, asked the very important question, In what way were they to differentiate between the relief given through the Poor Law and that given as pensions if public money were given as pensions to men who had not earned it? and he maintained that even if it were found possible to devise a practical scheme and set it to work immediately, something would have to be done to make the lot of the deserving poor better than it was at present in the workhouse. The question was, he maintained, far more urgent than many political questions which were now looked upon as pressing, and the difficulty would have to be met.

By drift of circumstances the old age pension question seems to be gradually getting reduced within narrower dimensions. It is being recognised that we have in fact, what is by so many people forgotten, a vast system of old age pension at present in operation under the Poor Law, and although that is a system which is in many places allowed to operate with a harshness which is born of its connection with the repressive portion of the Poor Law, there are sufficient examples to the contrary to show that it is capable of better things. On the other hand, it is being seen that even with the best of arrangements for old age pensions the guardians would not find themselves relieved of the care of the aged and infirm. If the granting of old age pensions would clear the field and leave the guardians nothing to do but to administer what may be termed the repressive side of the Poor Law, things would no doubt be much simplified. When we had given the pension we should be quit of all further responsibility. But a very small acquaintance with the sick and infirm wards of our workhouses is sufficient to make it plain enough that no amount of pension would give to the old and infirm the help and comfort which, even under the present *régime*, they now receive within some workhouses. It is but little recognised, we fear, by those who look upon old-age pensions as the panacea for all the discomforts of old age, that a very large number of the aged poor have outlived their relatives, and but for their workhouse associates would be absolutely friendless. Still more is it apt to be forgotten that the really old are helpless, forlorn, and often bed-ridden, and that they require far more nursing and attention than they could possibly obtain by means of any pension which any Government would be likely to offer them.

By co-operation in well-doing, as in the wards of a workhouse infirmary, such people can be provided, at a comparatively cheap rate, with comforts such as they would hardly dream of were they to endeavour to live out their lives upon their pensions. Thus we have it forcibly borne in upon us, and all the more forcibly the more infirmary wards we visit, that whatever may be done in regard to old-age pensions, the workhouse provision for the aged will have to go on side by side with it; and then comes the great question, Is it worth while to run two separate schemes concurrently for the relief of the aged, and does not the real solution of the problem lie rather in an improvement of existing arrangements—a much better classification of the inmates of our workhouses, a much greater liberty for the aged pensioners of the poor law, and a wide recognition of their *right* to the asylum offered to them, and of their independence of all pauper taint in accepting it? The fact is that the workhouse system was introduced as a repressive machinery to put down able-bodied pauperism. But the guardians have had forced upon them the further duty of providing a vast system of almshouses for the aged and hospitals for the sick. The latter task they have performed in some cases nobly, but too often with meagreness and parsimony, and the time seems to have arrived when functions so very opposite should be separated, and when the work of the taskmaster and of the good Samaritan should be put into different hands.

We do not think that we shall be wrong in anticipating that "classification" will be the keynote of the policy of the Local Government Board in regard to this double question of old age pensions and the treatment of the aged in the workhouses; nor can we doubt that if such classification should include a large extension of the infirmaries—so large an extension that they could take in the aged and infirm as well as the sick—and if in that way the sterner side of the workhouse system, as represented by the master, were utilised for the repression of pauperism, while the aged as well as the sick fell under the humaner influences which dealing with the sick tends especially to develop, a great reproach would be removed from the English method of treating our worn out workers.

